# Genomic Epidemiology Analysis of Infectious Disease Outbreaks Using TransPhylo

**DOI:** 10.1002/cpz1.60

**Published:** 2021-02-22

**Authors:** Xavier Didelot, Michelle Kendall, Yuanwei Xu, Peter J. White, Noel McCarthy

**Affiliations:** ^1^ School of Life Sciences and Department of Statistics University of Warwick United Kingdom; ^2^ Center for Computational Biology, Institute of Cancer and Genomic Sciences University of Birmingham United Kingdom; ^3^ Department of Infectious Disease Epidemiology, School of Public Health Imperial College London United Kingdom; ^4^ Medical Research Council Centre for Global Infectious Disease Analysis, School of Public Health Imperial College London United Kingdom; ^5^ National Institute for Health Research Health Protection Research Unit in Modelling and Health Economics, School of Public Health Imperial College London United Kingdom; ^6^ Modelling and Economics Unit, National Infection Service Public Health England London United Kingdom; ^7^ Warwick Medical School University of Warwick United Kingdom

**Keywords:** genomic epidemiology, infectious disease outbreak, phylogenetics, transmission analysis

## Abstract

Comparing the pathogen genomes from several cases of an infectious disease has the potential to help us understand and control outbreaks. Many methods exist to reconstruct a phylogeny from such genomes, which represents how the genomes are related to one another. However, such a phylogeny is not directly informative about transmission events between individuals. TransPhylo is a software tool implemented as an R package designed to bridge the gap between pathogen phylogenies and transmission trees. TransPhylo is based on a combined model of transmission between hosts and pathogen evolution within each host. It can simulate both phylogenies and transmission trees jointly under this combined model. TransPhylo can also reconstruct a transmission tree based on a dated phylogeny, by exploring the space of transmission trees compatible with the phylogeny. A transmission tree can be represented as a coloring of a phylogeny where each color represents a different host of the pathogen, and TransPhylo provides convenient ways to plot these colorings and explore the results. This article presents the basic protocols that can be used to make the most of TransPhylo. © 2021 The Authors.

**Basic Protocol 1**: First steps with TransPhylo

**Basic Protocol 2**: Simulation of outbreak data

**Basic Protocol 3**: Inference of transmission

**Basic Protocol 4**: Exploring the results of inference

## INTRODUCTION

Pathogen genomics has great potential to help us understand how infectious diseases spread between hosts. If we consider for example just two pathogen genomes from two separate cases, and we find that there are many differences between the genomes, then we can deduce that direct transmission is unlikely. Conversely, if there are few differences between the genomes, then transmission or a shared source is more likely. More generally, if we consider many genomes, each of which was isolated from a different case of the same disease, then we can ask ourselves what this data tells us about who infected whom among the individuals. We would not usually expect to be able to answer this question exactly, which is why it is important to use a statistical method of analysis that can correctly quantify all uncertainties.

Comparing many genomes of a pathogen is often done by constructing a phylogenetic tree, and the development of phylogenetic methods has a long and successful history (Yang & Rannala, 2012). In order to draw epidemiological interpretations, it is especially useful to consider a dated phylogeny (Biek, Pybus, Lloyd‐Smith, & Didelot, 2015; Drummond, Pybus, Rambaut, Forsberg, & Rodrigo, 2003; Grenfell et al., 2004). In a dated phylogeny, the axis represents time rather than genetic distance; the dates of the leaves are the known dates of sampling of the genomes and the dates of the internal nodes are the estimated dates when common ancestors existed. Reconstructing such a dated phylogeny requires a genome sequence alignment and the dates of at least some of the genomes. Software to reconstruct dated phylogenies include BEAST (Suchard et al., 2018), BEAST2 (Bouckaert et al., 2019), LSD (To, Jung, Lycett, & Gascuel, 2016), BactDating (Didelot, Croucher, Bentley, Harris, & Wilson, 2018), treedater (Volz & Frost, 2017), and TreeTime (Sagulenko, Puller, & Neher, 2018).

**Figure 1:**
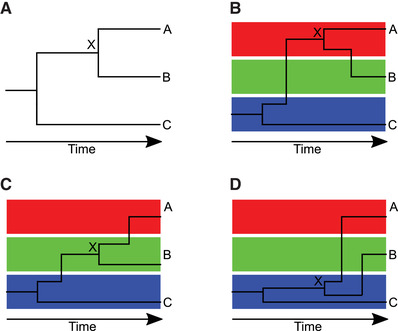
Illustration of how a given dated phylogeny (**A**) can be compatible with multiple transmission trees (**B‐D**). In parts (**B‐D**), the red, green and blue boxes correspond to evolution of the pathogen in hosts A, B, and C, respectively.

Dated phylogenies are extremely useful in genomic epidemiology, but it is important to note that they do not represent transmission events (Jombart, Eggo, Dodd, & Balloux, 2011; Pybus & Rambaut, 2009). Let us consider for example three hosts A, B ,and C (assumed to be the only cases of a disease), with the pathogen phylogeny showing A and B more closely related to one another than to C, as shown in Figure 1A. The node X corresponding to the last ancestor of A and B does not represent the point of transmission between A and B. This last ancestor X could have existed in host A, with transmission from A to B on the branch from X to B, as shown in Figure 1B. Or X could have existed in host B with transmission from B to A on the branch from X to A, as shown in Figure 1C. Or, it is even possible that X existed in host C with transmission from C to A on the branch from X to A and transmission from C to B on the branch from X to B, as shown in Figure 1D. Thus, the phylogeny indicates neither the timing nor the infector/infectee pairs of the transmission events, because the pathogen diversifies and evolves within each host (Didelot, Walker, Peto, Crook, & Wilson, 2016), and the phylogeny represents the sum of all this within‐host evolution.

Instead, the phylogeny can be thought of as the result of the within‐host evolution that happened in each of the sampled hosts and their infectors, and the infectors of their infectors, etc., going back to the source case from which all sampled cases were transmitted. This source case, like every infector in the transmission tree, may or may not be sampled. For each point in the phylogeny, we can ask which was the likely host of the pathogen: one of the sampled individuals, one of their infectors, one of the infectors of their infectors, etc. Answering this question is equivalent to breaking down the phylogeny into individual within‐host phylogenies. This formulation provides a direct bridge between phylogeny and transmission tree, since a jump of the pathogen from one host to another corresponds to a transmission event. TransPhylo was developed to solve exactly this problem: given a dated phylogeny, what can we infer about the transmission events between hosts? TransPhylo addresses this question by attempting to color the branches of the phylogeny with a different color for each host. If we assume that all cases have been sequenced, then we know that the number of colors must be equal to the number of leaves in the tree, with each leaf being of the color corresponding to each host (Didelot, Gardy, & Colijn, 2014). But we can also extend this coloring concept to the situation where some cases may not have been sampled, in which case additional colors can be used to represent unsampled cases that do not lead to any leaf (Didelot, Fraser, Gardy, & Colijn, 2017).

Basic Protocol 1 introduces the key concepts used in TransPhylo and Basic Protocol 2 demonstrates how to simulate outbreak data. Basic Protocol 3 shows how to infer a transmission tree given a dated phylogeny. This is the main functionality of TransPhylo, which most users are likely to want to follow and apply to their own data, but reading Basic Protocols 1 and 2 first will help users understand how Basic Protocol 3 works. Basic Protocol 4 presents several methods for exploring the results of a transmission tree inference, which will typically be applied after Basic Protocol 3.

## Basic Protocol 1 FIRST STEPS WITH TransPhylo

This protocol introduces the basic concepts used by TransPhylo. First the TransPhylo package is loaded, and then it is used to simulate and display both a transmission tree and corresponding phylogenetic tree.

### Necessary Resources

#### Hardware

The TransPhylo package is distributed as an R package within the Comprehensive R Archive Network (CRAN) project. It supports all computer platforms that can run R, including Windows (32‐bit, 64‐bit), Mac OS X (32‐bit, 64‐bit), and Unix/Linux.

#### Software

The latest TransPhylo stable release version for Windows, Mac OS X, and Unix/Linux is available from CRAN (https://cran.r‐project.org/package=TransPhylo). The latest development branch is available from GitHub (https://github.com/xavierdidelot/TransPhylo).

### Installing TransPhylo

1 Install R. The R statistical computing environment is required to run TransPhylo. Users need to download and install R from https://cran.r‐project.org
2 Install the TransPhylo package. Open R and type the following command:
**install.packages** ("TransPhylo")
  Alternatively, to install the latest development branch from GitHub you can use:
**install.packages** ("devtools")
devtools **::install_github** ("xavierdidelot/TransPhylo")
3 Load TransPhylo (which will also check that it is correctly installed) and check the version number using:
library(TransPhylo)
packageVersion("TransPhylo")

### Displaying an example, including both transmission tree and phylogenetic tree

**Figure 2:**
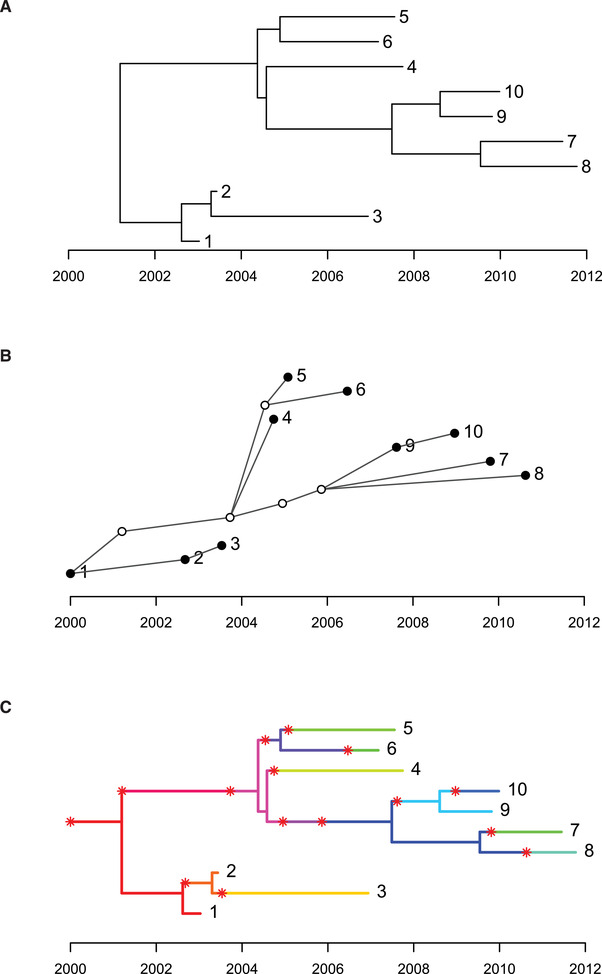
Dated phylogeny (**A**), transmission tree (**B**), and colored phylogeny (**C**) for a small simulated outbreak with ten sampled cases and five unsampled cases.

4 For illustration we use the code below which generates the three plots shown in Figure 2:
**set.seed** (0)
s= **simulateOutbreak** (nSampled=10)
pt= **extractPTree** (s)
**plot** (pt)
tt= **extractTTree** (s)
**plot** (tt)
**plot** (s)

After setting the random‐number generator seed so that the results are reproducible, the command simulateOutbreak is used to simulate an outbreak containing ten sampled cases. When simulating an outbreak, both the phylogenetic tree and the transmission tree are known exactly as described below. More details about outbreak simulation will be provided in Basic Protocol 2. This command returns an object of class ctree (stands for colored tree, for reasons which will become clear soon), which contains all the information about an outbreak. This information is essentially made of two inter‐dependent parts. The first part is the dated phylogeny representing how the ten sampled genomes are related to one another. The dated phylogeny can be extracted from a ctree object using the command extractPTree, which returns an object of class ptree (stands for phylogenetic tree), which can then be plotted as shown in Figure 2A. Each of the ten sampled genomes is shown as a leaf in this phylogeny, and is aligned on the *x* axis with the date when each of the ten genomes was isolated.

The second part of the simulated data is the transmission tree, which can be extracted from a ctree object using the command extractTTree. This returns an object of class ttree (stands for transmission tree), which can then be plotted as shown in Figure 2B. In this graphical representation of the transmission tree, each case is represented as a circle: the five empty circles represent unsampled cases and the filled circles represent the ten sampled cases. The presence of unsampled cases is due to the fact that TransPhylo does not assume that all cases in an outbreak are necessarily going to be reported and sequenced. Each circle is aligned on the *x* axis with the date when each case was infected. A link from one circle to another represents transmission from one case to another, which has to be in the direction from left to right since transmission from a case can only happen after infection of that case. We can see that the sampled individual labeled 1 was the source case of this outbreak, and that 1 infected 2, who in turn infected 3. Individual 1 also infected an unsampled individual around 2001 who infected another unsampled individual just before 2004, from which individual 4 was directly infected, as well as the remaining sampled cases via more unsampled cases. Note that each of the ten sampled cases shown by a filled circle in the transmission tree corresponds to one of the ten leaves of the phylogenetic tree, but the five unsampled cases shown by empty circles do not feature as leaves in the phylogenetic tree since by definition no genome was sampled from these cases. The transmission tree shown in Figure 2B includes the information about who infected whom, the date at which the transmission events happened, and the date at which sampling happened for the sampled cases.

### Relationship between the phylogenetic trees and the transmission tree

5 The phylogenetic tree and the transmission tree can be thought of as two sides of a same coin. When they are both known, as is the case here in a simulated dataset, these two aspects can be represented together on the same plot in the form of a colored tree (which is what the object class ctree stands for) as shown in Figure 2C. This colored tree concept is at the heart of how TransPhylo works. If we ignore the colors, then the tree is the same as the phylogenetic tree shown in Figure 2A. There is a unique color for each of the cases (sampled or unsampled). Each segment of the tree is colored according to the case that was hosting the pathogen. A change from one color to another, therefore, represents a transmission event, and these are highlighted with red stars. When a color reaches a leaf of the tree, it indicates that this case is sampled. If a color does not lead to any leaf, then it corresponds to an unsampled case. The dates of the stars, therefore, correspond to the dates of the transmission events as shown on the *x* axis of the transmission tree (Fig. 2B), and the colors before and after each star indicate who infected whom, as shown by the links in the transmission tree. This colored tree, therefore, contains all the information shown in both the phylogenetic tree (Fig. 2A) and the transmission tree (Fig. 2B). Note that the internal nodes of the phylogenetic tree do not correspond to transmission events, as is sometimes incorrectly assumed, and that instead transmission events (stars) can occur at any point along the branches of the phylogenetic tree.
  When simulating an outbreak as above (see Basic Protocol 2 for more details), both the phylogenetic tree and the transmission trees are known exactly. However, the main functionality of TransPhylo is to infer the transmission tree from the dated phylogeny. When performing such an inferential analysis (see Basic Protocol 3), the dated phylogeny is used as input and the transmission tree is the desired output. In other words, the phylogenetic tree is known in advance but only in black and white, and TransPhylo attempts to color the phylogeny to reveal the underlying transmission tree.

## Basic Protocol 2 SIMULATION OF OUTBREAK DATA

An outbreak can be simulated using the function simulateOutbreak. In Basic Protocol 1, this function was used without explanation of its various options, which are detailed here. The outbreak model in TransPhylo is a combination of a transmission model, a within‐host evolution model, and a sampling model, all of which can be parametrized as described below.

### Necessary Resources

#### Hardware

Windows (32‐bit, 64‐bit), Mac OS X (32‐bit, 64‐bit), or Unix/Linux computer

#### Software

The installation of TransPhylo was described in Basic Protocol 1

### Parameters of the transmission process

1 The outbreak is initiated with a single index case, who becomes infected at a time specified by the dateStartOutbreak parameter. The outbreak proceeds following a branching process, where each infected individual transmits to a number (possibly zero) of secondary cases, who in turn transmit to more individuals, etc. The first component of this transmission process is the offspring distribution, which represents how many secondary infections are caused by any case. A negative‐binomial distribution is used to represent this offspring distribution, with parameters off.r and off.p. The mean of this distribution is of special interest since it represents how many secondary infections are caused by each case on average. This value is often called the basic reproduction number and denoted *R*_0_, and it is equal to off.r*off.p/(1‐off.p). When the basic reproduction number is greater than 1, the outbreak is growing, whereas if it is lower than 1, the outbreak is shrinking. The second component of the branching process is the generation time distribution, defined here as the time interval between infection of an individual and subsequent transmission to another individual. A gamma distribution is used to represent this generation time distribution, with parameters w.shape and w.scale.

### Parameters of the within‐host evolution process

2 Within‐host evolution is assumed to follow a coalescent model with a constant population size. This model only has a single parameter neg, which represents the average time of coalescence of two lineages. We note that this within‐host model ignores the fact that the pathogen population size would normally grow within the host after infection, but it has the advantage of being simple both mathematically and computationally. A complete bottleneck is assumed at the point of transmission, such that a single variant from the within‐host population of the infector is selected uniformly at random to seed the new within‐host population of the infectee.

### Parameters of the sampling process

3 Sampling of cases is assumed to occur with probability pi for each case, and to happen at a time that is after the infection time by a value drawn from the sampling distribution. This distribution is modeled as a gamma distribution with parameters ws.shape and ws.scale. A limit to the sampling times can be imposed via the parameter dateT, in which case any individual with sampling time after this limit is effectively not sampled. This allows the simulation of ongoing outbreaks, where we know that there may be more cases reported beyond the current date, but alternatively the user can set dateT=Inf, which is equivalent to simulating a complete outbreak without any limit in time. If the outbreak is not finite, the simulation will not finish; there are various options to handle this, presented below.

### Simulation of an outbreak

4 The ten parameters described above (dateStartOutbreak, off.r, off.p, w.shape, w.scale, neg, pi, ws.shape, ws.scale and dateT) can all be specified when calling the function simulateOutbreak. If any of these parameters is not specified, then the following default values are assumed: dateStartOutbreak=2000, off.r=1, off.p=0.5, w.shape=2, w.scale=1, neg=0.25, pi=0.5, ws.shape=w.shape, ws.scale=w.scale , and dateT=Inf. Instead of the w.shape and w.scale parameters of the gamma distribution for the generation times, it is possible to specify the mean and standard deviation of this distribution via the parameters w.mean and w.std. Likewise, instead of the ws.shape and ws.scale parameters of the gamma distribution for the sampling times, it is possible to specify the mean and standard deviation of this distribution via the parameters ws.mean and ws.std.
  Finally, the optional parameter nSampled can be used to force the simulation to have a set number of sampled cases. This is achieved by repeating the simulation process until the desired number of sampled cases is reached. This is convenient, since otherwise the number of sampled cases can vary widely from one simulation to another. For illustration purposes, it is often useful to have a small but not trivial number of sampled cases, which is why nSampled=10 was used in Basic Protocol 1. However, it should be noted that a simulation thus obtained may be quite different from the simulation process without conditioning on the number of sampled cases. Depending on other parameters, the simulations may have a very small probability of having the desired number of sampled cases, in which case it could take a long time to repeat until this is achieved. In fact, the simulation process may not finish even without conditioning on the number of sampled cases, if the parameters are not carefully chosen. For example, simulateOutbreak(off.r=3) is likely not to finish, since it is attempting to simulate a complete outbreak (since by default dateT=Inf) with a basic reproduction number equal to 3 (since by default off.p=0.5, we have the basic reproduction number as off.r*off.p/(1‐off.p)=off.r). This has a very small chance of finishing early, for example if the index case did not cause any secondary infection, or if there was only one which itself did not cause any, etc. But in most instances the number of cases will grow exponentially when the basic reproduction number is >1, and the simulation will not finish. TransPhylo will attempt to simulate the whole outbreak even if it is not going to finish, and it is up to the user to set limits on how long the command is allowed to take, for example using the function setTimeLimit:
{ **setTimeLimit** (elapsed=5,transient=T)
s= **simulateOutbreak** (off.r=3)}

To simulate an outbreak with a basic reproduction number >1, it is therefore important to carefully set the other parameters to ensure that the number of infectees remains manageable. For example, let us consider a hypothetical pathogen for which the generation time is 1 year on average with a relatively small variance, which can be modeled using w.shape=10 and w.scale=0.1 (this corresponds to a mean w.mean=1 year and a standard deviation of w.std=0.316 years). We can simulate the first 3 years of the outbreak, from 2020 to 2023, using the code below:

**set.seed** (0)
s= **simulateOutbreak** (off.r=3,dateStartOutbreak=2020,dateT=2023,w.shape=10,w.scale=0.1)
**plot** ( **extractTTree** (s),type='detailed',w.shape=10,w.scale=0.1)
**plot** (s)

### Visualization of simulated outbreaks

**Figure 3:**
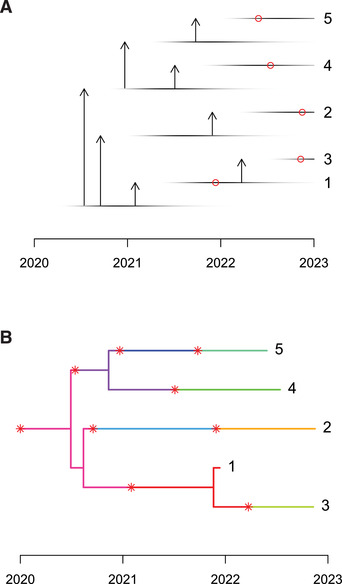
Detailed transmission tree (**A**) and colored phylogeny (**B**) for a small simulated outbreak.

5 The result is shown in Figure 3. The transmission tree (Fig. 3A) is shown here using an alternative representation from that previously used in Figure 2B, which captures some more details of the transmission process. There is a unique row for each individual in the transmission tree, whether they were sampled or not. The infectiousness of each individual is shown as a horizontal line with darkness proportional to the infectiousness. Sampling of individuals is shown by the red dots. In this example, there were nine cases in total, but only five were sampled. Both transmission (vertical arrows) and sampling (red dots) are more likely to happen when the infectiousness is high. This representation only shows the transmission tree, but the simulation also contains the phylogenetic tree of relationships between sampled genomes. Combining both the transmission tree and the phylogeny, we obtain a colored tree as explained in Basic Protocol 1, which is shown in Figure 3B.
  With a basic reproduction number equal to 3, the outbreak grows exponentially, which is not clear in the example above, but becomes obvious if we consider a longer time frame of, for example, 7 years with sampling of pi=0.1 of cases:
**set.seed** (0)
s= **simulateOutbreak** (off.r=3,dateStartOutbreak=2020,pi=0.1,dateT=2027,w.shape=10,w.scale=0.1)
**plot** ( **extractTTree** (s),showLabels=F)

**Figure 4:**
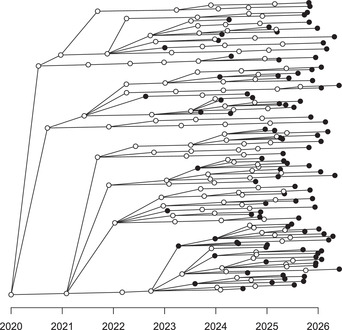
Transmission tree for a large simulated outbreak.

The resulting transmission tree is shown in Figure 4, where a more compact format is used to represent the tree due to the large number of cases involved. Specifically, there are 241 individuals represented in this transmission tree, 102 of which are sampled. Note that this proportion of sampling is higher than the specified value of the parameter pi for two reasons. Firstly, any individual who is unsampled and does not lead to at least one sampled individual is pruned from the transmission tree. Secondly, the outbreak is ongoing, so that recently infected individuals have little chance to satisfy the criteria in the previous sentence and are therefore pruned out. The reason for this pruning out is that transmission trees, as defined by the ttree class of TransPhylo, are designed to represent only sampled individuals and the unsampled individuals who acted as links between the sampled individuals. This is because the main aim of TransPhylo is to infer the transmission tree between sampled individuals (as described in detail in Basic Protocol 3). It is also interesting to note that few cases are shown in Figure 4 in the year 2026, even though the outbreak was simulated until the start of 2027. This happens because the sampling distribution (as determined by the parameters ws.shape and ws.scale) was not specified and therefore defaulted to the same distribution as the generation times (as determined by the parameters w.shape and w.scale) with mean 1 year and little variance. Individuals who became infected in 2026, therefore, have little chance to be sampled before the start of 2027, and therefore are not shown in the transmission tree.

### Exporting a simulated outbreak

6 This simulation protocol of TransPhylo is especially useful to simulate outbreaks and benchmark how accurate methods of inference (either Basic Protocol 3 of TransPhylo or some other method) are likely to be when applied to real datasets (Ness et al., 2019; Stimson et al., 2019; Walter et al., 2019). In this case, it is important to make sure that the parameters used for the simulation are realistic for the pathogen of interest in the real data. The dated phylogeny within a simulation can be saved for further use by first converting to the phylo class of the ape package (Paradis & Schliep, 2019), and then exporting to a file either in Newick or Nexus format using the following code:
**library** (ape)
phy= **phyloFromPTree** ( **extractPTree** (s))
**write.tree** (phy,file='tree.nwk')
**write.nexus** (phy,file='tree.nex')
**print** ( **dateLastSample** (s))

Both the Newick and Nexus formats store the dated phylogeny in relative time, so that the leaves and nodes are correctly spaced in time, but the absolute dates are unknown. However, subsequent analysis of the simulation may require knowing the absolute dates, especially in relation with the absolute dates when the outbreak started (parameter dateStartOutbreak) and when sampling of cases stopped (parameter dateT). It is then necessary to also store the absolute date of one of the samples, from which other dates can be deduced; TransPhylo expects the date of the last sample (or in other words the absolute date of the most recent leaf in the tree). In the case of the simulation from Figure 4, this date is 2026.994, which is measured in decimal years and falls just before the end of sampling at the start of 2027. If needed, dates in decimal years can be converted into calendar dates using the lubridate package (Grolemund & Wickham, 2011):

**library** (lubridate)
**format** ( **date_decimal** (2026.994), "%d‐%m‐%Y")

The date returned for the last sample is then the 29th December 2026, whereas the end of sampling at decimal year 2027.0 corresponds to 1st January 2027.

## Basic Protocol 3 INFERENCE OF TRANSMISSION

This basic protocol describes the main functionality of TransPhylo, which is to reconstruct a transmission tree from a dated phylogeny. The starting point for this protocol is a file containing the dated phylogeny. This would typically be reconstructed from dated genomes using BEAST (Suchard et al., 2018), BEAST2 (Bouckaert et al., 2019), or BactDating (Didelot et al., 2018). Here for simplicity and ease of reproducibility, we will use instead a dated phylogeny that was simulated using the script below:

**library** (ape)
**library** (TransPhylo)
**set.seed** (0)
s= **simulateOutbreak** (off.r=3,dateStartOutbreak=2020,dateT=2025,pi=0.3,w.shape=5,w.scale=0.3,neg=1)
**print** (s)
phy= **phyloFromPTree** ( **extractPTree** (s))
dls= **round** ( **dateLastSample** (s),digits=4) *#Returns 2024.9646*
phy **$** edge.length= **round** (phy **$** edge.length,digits=4)
**write.tree** (phy,file='input.nwk')
*#This file contains the Newick string used in the protocol below*

### Necessary Resources

#### Hardware

Windows (32‐bit, 64‐bit), Mac OS X (32‐bit, 64‐bit), or Unix/Linux computer

#### Software

The installation of TransPhylo was described in Basic Protocol 1

### Loading and displaying the phylogeny

1 The Newick representation of the tree, as well as the date of the last sample, which was 2024.9646, are used below to load and display this input tree:
**library** (ape)
**library** (TransPhylo)
tree= **read.tree** (text='((((3:2.5785,1:0.6433):0.1832,(10:1.7518,4:0.2621):0.9613):1.1929,((((9:1.4826,(2:0.2909,8:1.3392):0.1003):0.0811,13:2.2256):0.0165,6:1.6606):0.1252,7:1.2783):1.6824):0.2389,(11:4.3191,(12:3.0739,5:0.7795):0.3941):0.1049);')
p= **ptreeFromPhylo** (tree,dateLastSample = 2024.9646)
**plot** (p)

**Figure 5:**
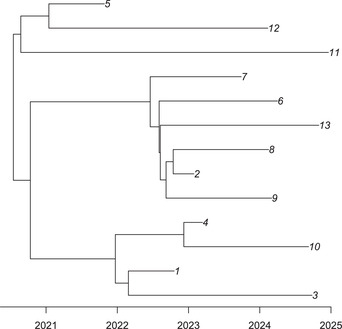
Dated phylogeny used as input for inference.

The result is shown in Figure 5. This tree contains 13 leaves, since in the simulation, there were 13 sampled individuals. This is only a fraction of the number of infected individuals, since the simulation used a sampling fraction of pi=0.3 and a date for the end of sampling of dateT=2025. The simulation also used a generation time distribution with parameters w.shape=5 and w.scale=0.3, a basic reproduction number of off.r=3 (since off.p=0.5 by default), and a within‐host coalescent time neg=1 year.

### Running the inference

2 Inference requires the date dateT at which sampling of cases ended, which is generally known for a given outbreak. If the outbreak has finished and is analyzed retrospectively, then we set dateT=Inf. If on the other hand the outbreak is ongoing and cases up to the current date are available, then the command decimal_date(now()) from the lubridate R package (Grolemund & Wickham, 2011) will provide the current date in decimal year format. For the analysis of the tree in Figure 5, we know that the simulation used dateT=2025.
  Inference also requires either the values w.shape and w.scale or w.mean and w.std for the Gamma distribution representing the generation time distribution. Here we will use the values that were used in the simulation, but when analyzing real datasets, it is necessary to estimate these parameters for the pathogen being analyzed. These parameters are available in the scientific literature for a wide range of pathogens. When the generation time distribution is not well documented, it can often be assumed to be approximately equal to the serial interval times between onset of symptom dates between pairs of infectors and infectees (Cori, Ferguson, Fraser, & Cauchemez, 2013). The distribution of interval between infection and sampling of a case also needs to be specified, and by default will be assumed to be equal to the generation time distribution. This should be approximately correct for many infectious diseases where symptoms are associated with both detection and transmission.
  To perform the inference, TransPhylo uses a method called Markov Chain Monte‐Carlo, which is often abbreviated as MCMC (Gilks, Richardson, & Spiegelhalter, 1996). Briefly, MCMC is an iterative procedure in which the transmission tree and other unknown parameters such as the sampling fraction pi are repetitively “guessed” and then discarded or retained according to how well they fit the data available. This is done in such a way that the values obtained during the course of the MCMC represent samples from the correct posterior distribution. It is important to run this procedure for long enough before starting to record the values visited to allow the MCMC to “converge,” i.e., to move away from the arbitrary chosen starting point of the MCMC and toward values that are probable in the posterior distribution. This initial phase of the MCMC is called the burn‐in. It is also necessary to run the MCMC for long enough after the burn‐in to allow the MCMC to “mix,” i.e., to thoroughly explore the range of plausible values. During the MCMC iterations after the burn‐in, the unknown parameters are recorded at regular intervals to return a manageable number of samples and to avoid autocorrelation between recorded values. The interval between two samples is called the thinning interval.
  Inference is performed as follows:
**set.seed** (0)
r= **inferTTree** (p,w.shape=5,w.scale=0.3,dateT=2025,mcmcIterations=1e5,thinning=10)
**plot** (r)

The first line sets the random‐number generator seed for reproducibility, the second line runs the inference, and the third line plots the result. In the second line, the first parameter p is the dated phylogeny formed in the previous script. The next three parameters specify the values of w.shape and w.scale for the generation time distribution, and the date dateT at which sampling of cases was stopped. The parameter mcmcIterations represents the number of MCMC iterations to perform in total (including burn‐in). Finally, the thinning interval is specified using the parameter thinning. Running this code on a standard laptop took approximately 2 min.

### Checking the convergence and mixing

**Figure 6:**
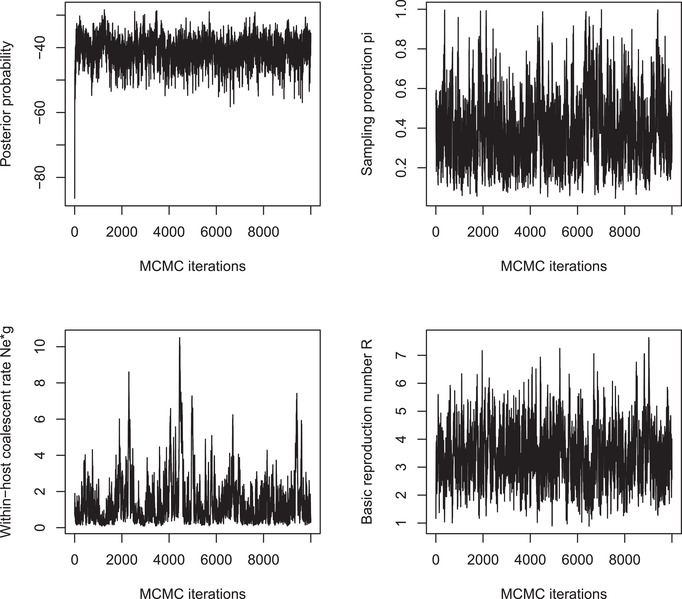
Traces of the Markov Chain Monte‐Carlo.

3 Before exploring the results of the inference (see Basic Protocol 4), it is necessary to make sure that the MCMC achieved good convergence and mixing properties. A first indication of this is given by plotting the traces of the MCMC, and this is what is done in the third line of the script above, which generates Figure 6. If the trace of each parameter looks like a caterpillar without any obvious upward or downward trend, as in Figure 6, it suggests that the MCMC has converged and mixed successfully. Otherwise, it may be necessary to increase the length of the MCMC.
  We recommend further inspection of the MCMC convergence and mixing using the CODA package (Plummer, Best, Cowles, & Vines, 2006), which implements several methods, one of which is to compute the Effective Sample Size (ESS) for each parameter. The ESS of each parameter needs to be at least 100, and can be computed as follows:
**install.packages** ("coda")
**library** (coda)
mcmc= **convertToCoda** (r)
**effectiveSize** (mcmc)

For the MCMC shown in Figure 6, we find that even the smallest ESS (for the within‐host coalescent time neg) is just above 100, which means that the mixing is satisfactory. We will explore this MCMC result in the Basic Protocol 4 below, in the interest of reproducibility of the results in a short amount of time. However, it should be noted that longer runs would be advisable to improve the MCMC mixing statistics, if this were an analysis of a real outbreak with the aim of sharing results. Longer runs can be performed by increasing the values of the parameters mcmcIterations. The run time of the command inferTTree is approximately proportional to the number of MCMC iterations, and therefore to the value of the parameter mcmcIterations. For example, a run ten times longer than the one above with parameters mcmcIterations=1e6,thinning=10 takes approximately 20 min and gives all ESS values above 500.

## Basic Protocol 4 EXPLORING THE RESULTS OF INFERENCE

In this protocol, we explore the results of the inference performed in the previous protocol, taking our starting point as r=inferTTree(…) as above and assuming that the convergence and mixing are satisfactory.

### Necessary Resources

#### Hardware

Windows (32‐bit, 64‐bit), Mac OS X (32‐bit, 64‐bit), or Unix/Linux computer

#### Software

The installation of TransPhylo was described in Basic Protocol 1

### Exploring the inferred parameter values

1 We start with the values of the main parameters, which can be displayed using:
**print** (r)

This command returns the mean and the 95% credible interval (in square brackets) of the main parameters in the inference. In the example, the command returns pi=0.41 [0.134;0.798], neg=1.30 [0.215;4.38], off.r=3.41 [1.71;5.61]. We note that the inferred values for these three parameters are close to the correct values used in the simulations (0.3, 1, and 3, respectively, see step 1). However, the credible intervals are quite large due to the fact that there is relatively little information about these parameters in this small dataset with just 13 leaves in the dated phylogeny (Fig. 5). Since the seed of the random number generator was set equal to zero in step 2 of Basic Protocol 3, the exact same values as above should be obtained when running the code above. However, if a different seed was used, slightly different values would be computed due to the Monte‐Carlo behavior of the algorithm.

### Exploring the inferred transmission tree

2 The inference also explores the probable possibilities for who infected whom. Each iteration of the MCMC explored one such transmission tree, but since it is not practical to visualize each of them separately, a statistical summary needs to be used. It is, however, difficult to summarize all the transmission trees in the posterior sample without losing information about their diversity. This problem is similar to the way Bayesian phylogenetic methods need to summarize their results (Heled & Bouckaert, 2013; Höhna, Landis, & Heath, 2017; Kendall & Colijn, 2016). One approach is to try to find a single transmission tree that best represents all the transmission trees in the posterior sample. TransPhylo can return such a tree by computing the medoid, which means finding the transmission tree from the posterior that is least different from all others according to a well‐defined distance metric (Kendall, Ayabina, Xu, Stimson, & Colijn, 2018). This can be achieved in TransPhylo using the following script:
med= **medTTree** (r)
**plot** (med)
**plot** ( **extractTTree** (med),type='detailed',w.shape=5,w.scale=0.3)

**Figure 7:**
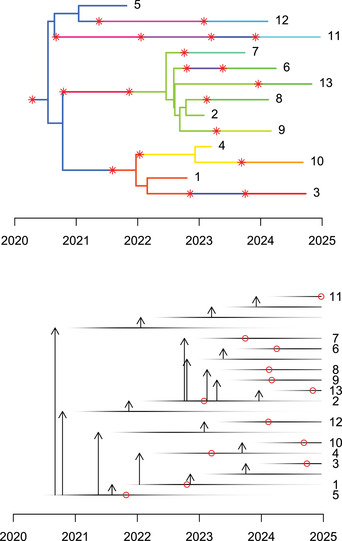
Medoid inferred transmission events shown as a colored phylogeny (**A**) and as a detailed transmission tree (**B**).

The result of this script is shown in Figure 7. The advantage of this approach is that it returns a single transmission tree, which can therefore be shown either as a coloring of the dated phylogeny (Fig. 7A) or as a separate transmission tree (Fig. 7B), using exactly the same visualization techniques as we described earlier for simulated transmission trees (Fig. 3A). However, the drawback of this approach is that it loses all information about the uncertainty of who infected whom. In real‐life applications, there are typically many transmission trees with non‐negligible statistical support, so that it is not possible to plot them all separately. A useful alternative is therefore to compute the probability of infection from each case to another. An extension of this idea is to compute the average distance from each case to another in number of transmission links. These two matrices can be computed and displayed, for example, using the lattice package (Sarkar, 2008) as follows:

**library** (lattice)
matWIW= **computeMatWIW** (r)
**levelplot** (matWIW)
matTDist= **computeMatTDist** (r)
**levelplot** (matTDist)

**Figure 8:**
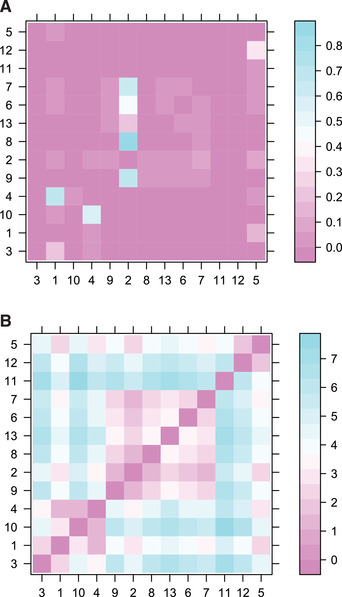
Matrix of transmission probabilities between cases (**A**) and matrix of distance between cases in the transmission tree (**B**).

The result of this script is shown in Figures 8A and 8B. Figure 8A shows that only five events have a posterior probability higher than 50%, namely the transmission events from 1 to 4, from 4 to 10, from 2 to 9, from 2 to 8, and from 2 to 7. Note that these five events are found in the medoid transmission tree (Fig. 7). The matrix matTDist is symmetric, as it measures the length of the transmission separating pairs of cases. Direct transmission corresponds to a distance of one, transmission via a single intermediate corresponds to a distance of two, etc. When this distance is one between cases A and B, the sum of the transmission probabilities from A to B and from B to A in the matrix matWIW is equal to one. However, the matrix matTDist provides more information about indirect transmission. For example, the transmission probabilities from A to B and from B to A would both be zero in matWIW regardless of whether there was a single individual or many intermediates in the transmission chain separating A and B.

### Exploring the inferred transmission properties of each host

3 The distributions of infection time and number of offspring for any individual can be computed and displayed using the following code, for example for the individual labeled ’1’:
tim= **getInfectionTimeDist** (r,k='1',show.plot = T)
off= **getOffspringDist** (r,k='1',show.plot = T)

**Figure 9:**
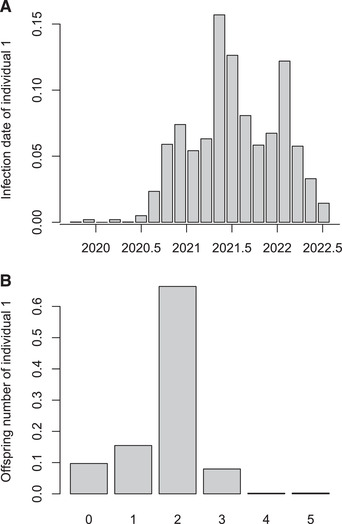
Infection date for a selected individual (**A**) and number of secondary cases caused by that same individual (**B**).

Figures 9A and 9B show the result of these two commands, respectively. Finally, we can compute the realized distributions in the transmission trees sampled by MCMC for the number of cases over time, the generation times, and the sampling times as follows:

**getIncidentCases** (r,show.plot = T)
**getGenerationTimeDist** (r,show.plot = T,maxi=4)
**getSamplingTimeDist** (r,show.plot = T,maxi=4)

**Figure 10:**
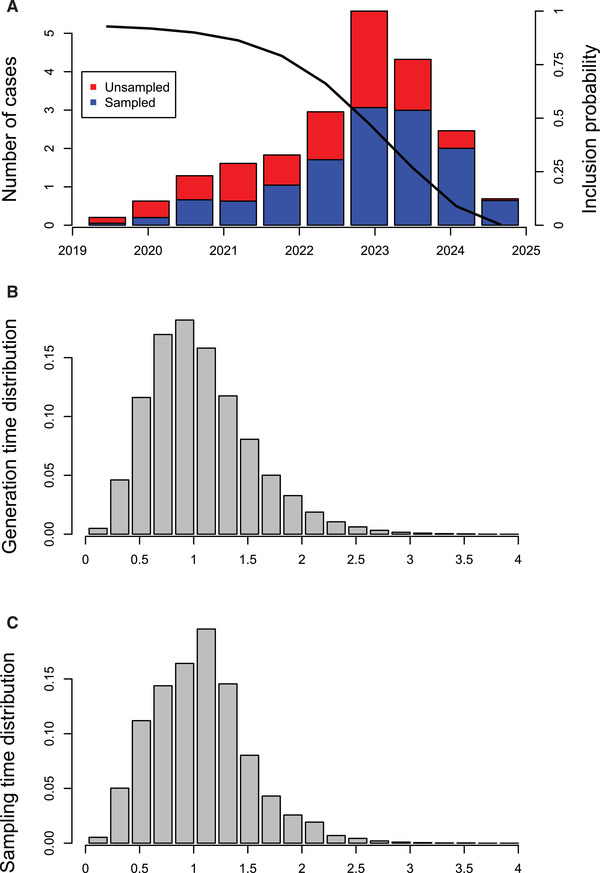
Number of cases in the transmission tree (**A**), realized generation time distribution (**B**), and realized sampling time distribution (**C**).

The output of these three commands is shown in Figure 10. In Figure 10A, the number of cases are shown over time against their infection dates (*x* axis) and colored according to whether they were sampled or not. Only the cases that feature in the transmission trees are shown, which includes all sampled cases plus unsampled cases that lead to at least one sampled case. This is the reason why the number of cases seems to go down in the last couple of years: individuals who have recently become infected are less likely to be sampled (due to the delay between infection and sampling) and also less likely to lead to another individual being sampled (due to both the delays from infection to onward transmission and from infection to sampling). Given these two effects, TransPhylo computes the probability that a case infected on a given date would feature in the transmission tree, and this is shown by the solid line in Figure 10A.

Figures 10B and 10C show the realized generation time distribution (delay from infection to transmission) and sampling distribution (delay from infection to sampling) for individuals that feature in the transmission tree. Both distributions are slightly shifted toward smaller values compared to the distributions used for simulation (which were both Gamma with a shape of 5 and scale of 0.3, and so have a mean of 1.5 years). This difference is expected, because individuals who have a smaller generation time and/or sampling time are more likely to be sampled (since sampling ended in 2025) or to have sampled descendants, and therefore more likely to be included in the transmission tree.

### Further exploration of inferred results

4 Depending on the user's interests, many further investigations can be performed on the TransPhylo results of inference of a transmission given a dated phylogeny. These results are fully stored in the object returned by the r=inferTTree(…) command. This object r has a relatively simple structure, which makes it easy for users to write their own code to explore it. It is simply a list with one element for each sampled MCMC iteration. If we consider the first such element, for example (r[[1]]), we can extract from it a full record of the state of the MCMC at that iteration, including the colored tree (r[[1]]$ctree) and the values of the parameters neg, pi, off.r and off.p (r[[1]]$neg, r[[1]]$pi, r[[1]]$off.r, r[[1]]$off.p, respectively).

## COMMENTARY

### Background Information

A simple method for assessing the probability of transmission between two individuals is to count the number of differences between two pathogen isolate genomes, and to rule out transmission if this number is greater than a carefully chosen threshold (Eyre et al., 2013; Walker et al., 2013, 2014). It is, however, difficult to determine what the threshold should be. A slightly more natural approach is to estimate the time of the most recent common ancestor between the two genomes, in order to assess whether this ancestor could have lived within one of the individuals who would have infected the other one, which can more easily be assessed based on pathogen epidemiology (Didelot et al., 2012b, 2013; Eldholm et al., 2016). The simplicity of these pairwise approaches is attractive, and they make little assumption about epidemiological factors, such as whether or not all cases were sampled. However, a pairwise approach is inherently limited: the distance between genomes from A and B may tell us something different about transmission between A and B once we account for their distances to other genomes.

In order to avoid this problem with pairwise approaches, it is necessary to consider the whole transmission tree in a single analysis. One of the earliest methods of transmission tree reconstruction was SeqTrack (Jombart et al., 2011), which later evolved into outbreaker (Campbell et al., 2018; Jombart et al., 2014). The main contribution of TransPhylo compared to these methods is that it accounts for the within‐host evolution when reconstructing a transmission tree, which can be very important to avoid overinterpretation of the genomic data (Didelot et al., 2016). The first version of TransPhylo was written in Matlab and assumed that all cases were sampled (Didelot et al., 2014). This version was therefore only useful in the rare cases where field epidemiologists were certain to have detected and sequenced all or almost all cases (Hatherell et al., 2016). Alternative approaches that can jointly infer the transmission tree and phylogeny also require full sampling (Hall, Woolhouse, & Rambaut, 2015; Klinkenberg, Backer, Didelot, Colijn, & Wallinga, 2017; Ypma, van Ballegooijen, & Wallinga, 2013). More recently, TransPhylo has been rewritten in R and can handle both unsampled cases and ongoing outbreaks (Didelot et al., 2017).

TransPhylo has been applied to a large number of different pathogens, including tuberculosis outbreaks in various settings (Ayabina et al., 2018; Folkvardsen et al., 2017; Khan et al., 2019; Séraphin et al., 2018; Xu et al., 2019; Xu et al., 2020; Yang et al., 2018), HIV (Mak et al., 2020; Ratmann et al., 2017), mumps (Stapleton et al., 2019), *Staphylococcus aureus* (Cheng et al., 2019), *Klebsiella pneumoniae* (Kwong et al., 2018; van Dorp et al., 2019), *Neisseria gonorrhoeae* (Osnes et al., 2020; Whittles, White, & Didelot, 2019), and SARS‐CoV‐2 (Mavian, Marini, Prosperi, & Salemi, 2020; Wang et al., 2020).

### Critical Parameters

When inferring a transmission tree using TransPhylo, it is important that the phylogenetic tree used as input be correctly reconstructed and dated. As much genetic data per isolate as possible should be used for the phylogenetic inference, and ideally whole genome sequences should be used since this provides the best genetic resolution (Didelot, Bowden, Wilson, Peto, & Crook, 2012a). For the dating, it is important to carefully consider the choice of molecular clock model that relates genetic distances with time (Didelot, Siveroni, & Volz, 2021). The parameters w.shape and w.scale specifying the generation time distribution should also be carefully chosen based on the epidemiology of the infectious pathogen under study, as described in Basic Protocol 3. When there is considerable uncertainty about these parameters and/or the phylogeny, sensitivity analysis can be used to test the robustness of the transmission analysis results (Didelot et al., 2014; Xu et al., 2019, 2020).

### Troubleshooting

The protocols should be easily reproducible if applied using exactly the same conditions as described above. However, problems can arise if the conditions are changed, for example if inferring the transmission tree (Basic Protocol 3) based on a new set of pathogen genomes. It is often possible to diagnose and solve the problem by breaking down the process into small steps or perfoming the analysis for selected subsets of the data. Several frequently occurring problems with solutions are described at https://github.com/xavierdidelot/TransPhylo/issues, which is also the best place to report new issues and ask for help from the authors and wider TransPhylo community.

### Understanding Results

The first step to ensure that results of a TransPhylo transmission analysis are correctly understood is to check the convergence and mixing properties of the TransPhylo algorithm, as described in Basic Protocol 3. If the MCMC has not converged or mixed appropriately, the results will be meaningless, and it is therefore necessary to re‐run the algorithm with more iterations. Once good convergence and mixing properties have been achieved, the results can be interpreted following the steps described in Basic Protocol 4. It is important to bear in mind that TransPhylo does not reconstruct a single transmission tree, even if methods are described to summarize the results into this format for convenience. Instead, TransPhylo explores the whole posterior distribution of transmission trees, which typically includes significant uncertainty on at least some of the exact transmission links.

### Time Considerations

The time taken to run a TransPhylo transmission analysis is determined by the number of iterations being done in the MCMC, which needs to be large enough to achieve good convergence and mixing properties. The analysis of a typical dataset (∼100 genomes with a sampling proportion around 50%) requires around a million iterations, which takes between one and a few hours (Didelot et al., 2017). For larger datasets, the run time should be approximately proportional to the number of genomes, all other factors being equal. The analysis of more sparsely sampled datasets is expected to take longer than the analysis of more densely sampled datasets, for two reasons. Firstly, the likelihood computation performed at each step of the MCMC takes time that is proportional to the number of cases (sampled or unsampled) in the transmission tree. Secondly, the number of iterations required also increases with the sparsity of the sampling, since this implies more unknown quantities to infer for which the uncertainty needs to be quantified.

### Author Contributions

**Xavier Didelot**: writing‐original draft; writing‐review & editing. **Michelle Kendall**: writing‐original draft; writing‐review & editing. **Yuanwei Xu**: writing‐original draft; writing‐review & editing. **Peter J. White**: writing‐original draft; writing‐review & editing. **Noel McCarthy**: writing‐original draft; writing‐review & editing.

## References

[cpz160-bib-0001] Ayabina, D. , Ronning, J. O. , Alfsnes, K. , Debech, N. , Brynildsrud, O. B. , Arnesen, T. , … Eldholm, V. (2018). Genome‐based transmission modelling separates imported tuberculosis from recent transmission within an immigrant population. Microbial Genomics, 4, e000219. doi: 10.1099/mgen.0.000219. PMC6249437 30216147

[cpz160-bib-0002] Biek, R. , Pybus, O. G. , Lloyd‐Smith, J. O. , & Didelot, X. (2015). Measurably evolving pathogens in the genomic era. Trends in Ecology & Evolution, 30, 306–313. 25887947 10.1016/j.tree.2015.03.009 PMC4457702

[cpz160-bib-0003] Bouckaert, R. , Vaughan, T. G. , Fourment, M. , Gavryushkina, A. , Heled, J. , Denise, K. , … Drummond, A. J. (2019). BEAST 2.5 : An advanced software platform for Bayesian Evolutionary Analysis. PLoS Computational Biology, 15, e1006650. doi: 10.1371/journal.pcbi.1006650. 30958812 PMC6472827

[cpz160-bib-0004] Campbell, F. , Didelot, X. , Fitzjohn, R. , Ferguson, N. M. , Cori, A. , & Jombart, T. (2018). Outbreaker2: A modular platform for outbreak reconstruction. BMC Bioinformatics, 19, 363. doi: 10.1186/s12859-018-2330-z. 30343663 PMC6196407

[cpz160-bib-0005] Cheng, V. C. C. , Wong, S. C. , Cao, H. , Chen, J. H. K. , So, S. Y. C. , Wong, S. C. Y. , … Ho, P. L. (2019). Whole‐genome sequencing data‐based modeling for the investigation of an outbreak of community‐associated methicillin‐resistant *Staphylococcus aureus* in a neonatal intensive care unit in Hong Kong. European Journal of Clinical Microbiology and Infectious Diseases, 38, 563–573. doi: 10.1007/s10096-018-03458-y. 30680562

[cpz160-bib-0006] Cori, A. , Ferguson, N. M. , Fraser, C. , & Cauchemez, S. (2013). A new framework and software to estimate time‐varying reproduction numbers during epidemics. American journal of epidemiology, 178, 1505–1512. doi: 10.1093/aje/kwt133. 24043437 PMC3816335

[cpz160-bib-0007] Didelot, X. , Bowden, R. , Wilson, D. J. , Peto, T. E. A. , & Crook, D. W. (2012a). Transforming clinical microbiology with bacterial genome sequencing. Nature Reviews Genetics, 13, 601–612. doi: 10.1038/nrg3226. PMC5049685 22868263

[cpz160-bib-0008] Didelot, X. , Croucher, N. J. , Bentley, S. D. , Harris, S. R. , & Wilson, D. J. (2018). Bayesian inference of ancestral dates on bacterial phylogenetic trees. Nucleic Acids Research, 46, e134. doi: 10.1093/nar/gky783. 30184106 PMC6294524

[cpz160-bib-0009] Didelot, X. , Eyre, D. W. , Cule, M. , Ip, C. L. C. , Ansari, M. A. , Griffiths, D. , … Harding, R. M. (2012b). Microevolutionary analysis of *Clostridium difficile* genomes to investigate transmission. Genome Biology, 13, R118. doi: 10.1186/gb-2012-13-12-r118. 23259504 PMC4056369

[cpz160-bib-0010] Didelot, X. , Fraser, C. , Gardy, J. , & Colijn, C. (2017). Genomic infectious disease epidemiology in partially sampled and ongoing outbreaks. Molecular Biology and Evolution, 34, 997–1007. 28100788 10.1093/molbev/msw275 PMC5850352

[cpz160-bib-0011] Didelot, X. , Gardy, J. , & Colijn, C. (2014). Bayesian inference of infectious disease transmission from whole genome sequence data. Molecular Biology and Evolution, 31, 1869–1879. doi: 10.1093/molbev/msu121. 24714079 PMC4069612

[cpz160-bib-0012] Didelot, X. , Nell, S. , Yang, I. , Woltemate, S. , van der Merwe, S. , & Suerbaum, S. (2013). Genomic evolution and transmission of *Helicobacter pylori* in two South African families. Proceedings of the National Academy of Sciences of the United States of America, 110, 13880–13885. doi: 10.1073/pnas.1304681110. 23898187 PMC3752273

[cpz160-bib-0013] Didelot, X. , Siveroni, I. , & Volz, E. M. (2021). Additive uncorrelated relaxed clock models for the dating of genomic epidemiology phylogenies. Molecular Biology and Evolution, 38, 307–317. doi: 10.1093/molbev/msaa193. 32722797 PMC8480190

[cpz160-bib-0014] Didelot, X. , Walker, A. S. , Peto, T. E. , Crook, D. W. , & Wilson, D. J. (2016). Within‐host evolution of bacterial pathogens. Nature Reviews Microbiology, 14, 150–162. doi: 10.1038/nrmicro.2015.13. 26806595 PMC5053366

[cpz160-bib-0015] van Dorp, L. , Wang, Q. , Shaw, L. P. , Acman, M. , Brynildsrud, O. B. , Eldholm, V. , … Wang, H. (2019). Rapid phenotypic evolution in multidrug‐resistant *Klebsiella pneumoniae* hospital outbreak strains. Microbial Genomics, 5, e000263. doi: 10.1099/mgen.0.000263. PMC6521586 30939107

[cpz160-bib-0016] Drummond, A. J. , Pybus, O. G. , Rambaut, A. , Forsberg, R. , & Rodrigo, A. G. (2003). Measurably evolving populations. Trends in Ecology and Evolution, 18, 481–488. doi: 10.1016/S0169-5347(03)00216-7.

[cpz160-bib-0017] Eldholm, V. , Rieux, A. , Monteserin, J. , Lopez, J. M. , Palmero, D. , Lopez, B. , … Balloux, F. (2016). Impact of HIV co‐infection on the evolution and transmission of multidrug‐resistant tuberculosis. eLife, 5, e16644. doi: 10.7554/eLife.16644. 27502557 PMC4978521

[cpz160-bib-0018] Eyre, D. W. , Cule, M. L. , Wilson, D. J. , Griffiths, D. , Vaughan, A. , O'Connor, L. , … Walker, A. S. (2013). Diverse sources of *C. difficile* infection identified on whole‐genome sequencing. New England Journal of Medicine, 369, 1195–1205. doi: 10.1056/NEJMoa1216064. PMC3868928 24066741

[cpz160-bib-0019] Folkvardsen, D. B. , Norman, A. , Andersen, Å. B. , Rasmussen, E. M. , Jelsbak, L. , & Lillebaek, T. (2017). Genomic epidemiology of a major *Mycobacterium tuberculosis* outbreak: Retrospective cohort study in a low‐incidence setting using sparse time‐series sampling. Journal of Infectious Diseases, 216, 366–374. doi: 10.1093/infdis/jix298. 28666374

[cpz160-bib-0020] Gilks, W. R. , Richardson, S. , & Spiegelhalter, D. J. (Eds.) (1996). Markov chain Monte Carlo in practice. London, UK: Chapman & Hall/CRC.

[cpz160-bib-0021] Grenfell, B. T. , Pybus, O. G. , Gog, J. R. , Wood, J. L. N. , Daly, J. M. , Mumford, J. A , & Holmes, E. C. (2004). Unifying the epidemiological and evolutionary dynamics of pathogens. Science, 303, 327–332. doi: 10.1126/science.1090727. 14726583

[cpz160-bib-0022] Grolemund, G. , & Wickham, H. (2011). Dates and times made easy with lubridate. Journal of Statistical Software, 40, 1–25. doi: 10.18637/jss.v040.i03.

[cpz160-bib-0023] Hall, M. , Woolhouse, M. , & Rambaut, A. (2015). Epidemic reconstruction in a phylogenetics framework: Transmission trees as partitions of the node set. PLoS Computational Biology, 11, e1004613. doi: 10.1371/journal.pcbi.1004613. 26717515 PMC4701012

[cpz160-bib-0024] Hatherell, H.‐A. , Didelot, X. , Pollock, S. L. , Tang, P. , Crisan, A. , Johnston, J. C. , … Gardy, J. (2016). Declaring a tuberculosis outbreak over with genomic epidemiology. Microbial Genomics, 2(5), e000060. doi: 10.1099/mgen.0.000060. 28348853 PMC5320671

[cpz160-bib-0025] Heled, J. , & Bouckaert, R. R. (2013). Looking for trees in the forest: Summary tree from posterior samples. BMC Evolutionary Biology, 13, 221. doi: 10.1186/1471-2148-13-221. 24093883 PMC3853548

[cpz160-bib-0026] Höhna, S. , Landis, M. J. , & Heath, T. A. (2017). Phylogenetic inference using RevBayes. Current Protocols in Bioinformatics, 2017, 6.16.1–6.16.34. 10.1002/cpbi.22 28463399

[cpz160-bib-0027] Jombart, T. , Cori, A. , Didelot, X. , Cauchemez, S. , Fraser, C. , & Ferguson, N. (2014). Bayesian reconstruction of disease outbreaks by combining epidemiologic and genomic data. PLoS Computational Biology, 10, e1003457. doi: 10.1371/journal.pcbi.1003457. 24465202 PMC3900386

[cpz160-bib-0028] Jombart, T. , Eggo, R. M. , Dodd, P. J. , & Balloux, F. (2011). Reconstructing disease outbreaks from genetic data: A graph approach. Heredity, 106, 383–390. doi: 10.1038/hdy.2010.78. 20551981 PMC3183872

[cpz160-bib-0029] Kendall, M. , Ayabina, D. , Xu, Y. , Stimson, J. , & Colijn, C. (2018). Estimating transmission from genetic and epidemiological data: A metric to compare transmission trees. Statistical Science, 33, 70–85. doi: 10.1214/17-STS637.

[cpz160-bib-0030] Kendall, M. , & Colijn, C. (2016). Mapping phylogenetic trees to reveal distinct patterns of evolution. Molecular Biology and Evolution, 33, 2735–2743. doi: 10.1093/molbev/msw124. 27343287 PMC5026250

[cpz160-bib-0031] Khan, P. Y. , Yates, T. A. , Osman, M. , Warren, R. M. , van der Heijden, Y. , Padayatchi, N. , … Pym, A. (2019). Transmission of drug‐resistant tuberculosis in HIV‐endemic settings. The Lancet Infectious Diseases, 19, e77–e88. doi: 10.1016/S1473-3099(18)30537-1. 30554996 PMC6474238

[cpz160-bib-0032] Klinkenberg, D. , Backer, J. A. , Didelot, X. , Colijn, C. , & Wallinga, J. (2017). Simultaneous inference of phylogenetic and transmission trees in infectious disease outbreaks. PLoS Computational Biology, 13, e1005495. doi: 10.1371/journal.pcbi.1005495. 28545083 PMC5436636

[cpz160-bib-0033] Kwong, J. C. , Lane, C. R. , Romanes, F. , Gonçalves da Silva, A. , Easton, M. , Cronin, K. , … Howden, B. P. (2018). Translating genomics into practice for real‐time surveillance and response to carbapenemase‐producing Enterobacteriaceae: Evidence from a complex multi‐institutional KPC outbreak. PeerJ, 6, e4210. doi: 10.7717/peerj.4210. 29312831 PMC5756455

[cpz160-bib-0034] Mak, L. , Perera, D. , Lang, R. , Kossinna, P. , He, J. , Gill, M. J. , … van Marle, G. (2020). Evaluation of a phylogenetic pipeline to examine transmission networks in a Canadian HIV cohort. Microorganisms, 8, 1–17. doi: 10.3390/microorganisms8020196. PMC7074708 32023939

[cpz160-bib-0035] Mavian, C. , Marini, S. , Prosperi, M. , & Salemi, M. (2020). A snapshot of SARS‐CoV‐2 genome availability up to April 2020 and its implications: Data analysis. JMIR Public Health and Surveillance, 6, e19170. doi: 10.2196/19170. 32412415 PMC7265655

[cpz160-bib-0036] Ness, S. E. Van , Lee, R. , Sebastiani, P. , Horsburgh, C. R. , Jenkins, H. E. , & White, L. F. (2019). Estimating the relative probability of direct transmission between infectious disease patients. bioRxiv, 612945. doi: 10.1101/612945. PMC7394954 32211747

[cpz160-bib-0037] Osnes, M. N. , Didelot, X. , de Korne‐Elenbaas, J. , Alfsnes, K. , Brynildsrud, O. B. , Syversen, G. , … Eldholm, V. (2020). Sudden emergence of a Neisseria gonorrhoeae clade with reduced susceptibility to extended‐spectrum cephalosporins, Norway. Microbial Genomics, 6(12). doi: 10.1099/mgen.0.000480. PMC8116678 33200978

[cpz160-bib-0038] Paradis, E. , & Schliep, K. (2019). Ape 5.0: An environment for modern phylogenetics and evolutionary analyses in R. Bioinformatics, 35, 526–528. doi: 10.1093/bioinformatics/bty633. 30016406

[cpz160-bib-0039] Plummer, M. , Best, N. , Cowles, K. , & Vines, K. (2006). CODA: Convergence diagnosis and output analysis for MCMC. R News, 6, 7–11.

[cpz160-bib-0040] Pybus, O. G. , & Rambaut, A. (2009). Evolutionary analysis of the dynamics of viral infectious disease. Nature Reviews Genetics, 10, 540–550. doi: 10.1038/nrg2583. PMC7097015 19564871

[cpz160-bib-0041] Ratmann, O. , Hodcroft, E. B. , Pickles, M. , Cori, A. , Hall, M. , Lycett, S. , … Fraser, C. (2017). Phylogenetic tools for generalized HIV‐1 epidemics: Findings from the PANGEA‐HIV methods comparison. Molecular Biology and Evolution, 34, 185–203. doi: 10.1093/molbev/msw217. 28053012 PMC5854118

[cpz160-bib-0042] Sagulenko, P. , Puller, V. , & Neher, R. A. (2018). TreeTime: Maximum likelihood phylodynamic analysis. Virus Evolution, 4, vex042. doi: 10.1093/ve/vex042. 29340210 PMC5758920

[cpz160-bib-0043] Sarkar, D. (2008). Lattice: nultivariate data visualization with R. New York: Springer.

[cpz160-bib-0044] Séraphin, M. N. , Didelot, X. , Nolan, D. J. , May, J. R. , Khan, M. S. R. , Murray, E. R. , … Lauzardo, M. (2018). Genomic investigation of a mycobacterium tuberculosis outbreak involving prison and community cases in Florida, United States. American Journal of Tropical Medicine and Hygiene, 99, 867–874. doi: 10.4269/ajtmh.17-0700. PMC6159577 29987998

[cpz160-bib-0045] Stapleton, P. J. , Eshaghi, A. , Seo, C. Y. , Wilson, S. , Harris, T. , Deeks, S. L. , … Patel, S. N. (2019). Evaluating the use of whole genome sequencing for the investigation of a large mumps outbreak in Ontario, Canada. Scientific Reports, 9, 1–11. doi: 10.1038/s41598-019-47740-1. 31471545 PMC6717193

[cpz160-bib-0046] Stimson, J. , Gardy, J. , Mathema, B. , Crudu, V. , Cohen, T. , & Colijn, C. (2019). Beyond the SNP threshold: Identifying outbreak clusters using inferred transmissions. Molecular Biology and Evolution, 36, 587–603. doi: 10.1093/molbev/msy242. 30690464 PMC6389316

[cpz160-bib-0047] Suchard, M. A. , Lemey, P. , Baele, G. , Ayres, D. L. , Drummond, A. J. , & Rambaut, A. (2018). Bayesian phylogenetic and phylodynamic data integration using BEAST 1.10. Virus Evolution, 4, vey016. doi: 10.1093/ve/vey016. 29942656 PMC6007674

[cpz160-bib-0048] To, T.‐H. , Jung, M. , Lycett, S. , & Gascuel, O. (2016). Fast dating using least‐squares criteria and algorithms. Systematic Biology, 65, 82–97. doi: 10.1093/sysbio/syv068. 26424727 PMC4678253

[cpz160-bib-0049] Volz, E. M. , & Frost, S. D. W. (2017). Scalable relaxed clock phylogenetic dating. Virus Evolution, 3, vex025. doi: 10.1093/ve/vex025.

[cpz160-bib-0050] Walker, T. M. , Ip, C. L. C. , Harrell, R. H. , Evans, J. T. , Kapatai, G. , Dedicoat, M. J. , … Peto, T. E. (2013). Whole‐genome sequencing to delineate *Mycobacterium tuberculosis* outbreaks: A retrospective observational study. The Lancet Infectious Diseases, 13, 137–146. doi: 10.1016/S1473-3099(12)70277-3. 23158499 PMC3556524

[cpz160-bib-0051] Walker, T. M. , Lalor, M. K. , Broda, A. , Ortega, L. S. , Morgan, M. , Parker, L. , … Conlon, C. P. (2014). Assessment of *Mycobacterium tuberculosis* transmission in Oxfordshire, UK, 2007–12, with whole pathogen genome sequences: An observational study. The Lancet Respiratory Medicine, 2, 285–292. doi: 10.1016/S2213-2600(14)70027-X. 24717625 PMC4571080

[cpz160-bib-0052] Walter, K. S. , Colijn, C. , Cohen, T. , Mathema, B. , Liu, Q. , Bowers, J. , & David, M. (2019). Genomic variant identification methods alter *Mycobacterium tuberculosis* transmission inference. bioRxiv, 1–28. 10.1099/mgen.0.000418 PMC7641424 32735210

[cpz160-bib-0053] Wang, L. , Didelot, X. , Yang, J. , Wong, G. , Shi, Y. , Liu, W. , … Bi, Y. (2020). Inference of person‐to‐person transmission of COVID‐19 reveals hidden super‐spreading events during the early outbreak phase. Nature Communications, 11, 1–6. 10.1038/s41467-020-18836-4 PMC7538999 33024095

[cpz160-bib-0054] Whittles, L. K. , White, P. J. , & Didelot, X. (2019). A dynamic power‐law sexual network model of gonorrhoea outbreaks. PLoS Computational Biology, 15, e1006748. doi: 10.1371/journal.pcbi.1006748. 30849080 PMC6426262

[cpz160-bib-0055] Xu, Y. , Cancino‐Munoz, I. , Torres‐Puente, M. , Villamayor, L. M. , Borrás, R. , Borrás‐Máñez, M. , … Comas, I. (2019). High‐resolution mapping of tuberculosis transmission: Whole genome sequencing and phylogenetic modelling of a cohort from Valencia Region, Spain. PLoS Medicine, 16, 1–20. doi: 10.1371/journal.pmed.1002961. PMC6822721 31671150

[cpz160-bib-0056] Xu, Y. , Stockdale, J. E. , Naidu, V. , Hatherell, H. , Stimson, J. , Stagg, H. R. , … Colijn, C. (2020). Transmission analysis of a large tuberculosis outbreak in London: A mathematical modelling study using genomic data. Microbial Genomics, 6, 761411. doi: 10.1099/mgen.0.000450. PMC7725332 33174832

[cpz160-bib-0057] Yang, C. , Lu, L. , Warren, J. L. , Wu, J. , Jiang, Q. , Zuo, T. , … Cohen, T. (2018). Internal migration and transmission dynamics of tuberculosis in Shanghai, China: An epidemiological, spatial, genomic analysis. The Lancet Infectious Diseases, 18, 788–795. doi: 10.1016/S1473-3099(18)30218-4. 29681517 PMC6035060

[cpz160-bib-0058] Yang, Z. , & Rannala, B. (2012). Molecular phylogenetics: Principles and practice. Nature Reviews Genetics, 13, 303–314. doi: 10.1038/nrg3186. 22456349

[cpz160-bib-0059] Ypma, R. , van Ballegooijen, W. M. , & Wallinga, J. (2013). Relating phylogenetic trees to transmission trees of infectious disease outbreaks. Genetics, 195, 1055–1062. doi: 10.1534/genetics.113.154856. 24037268 PMC3813836

[cpz160-bib-0060] https://CRAN.R-project.org/package=TransPhylo.

[cpz160-bib-0061] https://github.com/xavierdidelot/TransPhylo.

[cpz160-bib-0062] http://xavierdidelot.github.io/TransPhylo/.

